# Characteristics of adverse drug reactions in a vemurafenib early post-marketing phase vigilance study in Japan

**DOI:** 10.1007/s12094-017-1706-2

**Published:** 2017-07-03

**Authors:** H. Uhara, Y. Kiyohara, A. Tsuda, M. Takata, N. Yamazaki

**Affiliations:** 1 Department of Dermatology, Shinshu University School of Medicine, Nagano, Japan; 2 Department of Dermatology, Sapporo Medical University School of Medicine, South 1, West 16, Chuo-ku, Sapporo, Hokkaido 060-8556 Japan; 3 Dermatology Division, Shizuoka Cancer Center Hospital, Shizuoka, Japan; 4 Chugai Pharmaceutical Co., Ltd., Tokyo, Japan; 5 Department of Dermatologic Oncology, National Cancer Center Hospital, Tokyo, Japan

**Keywords:** Vemurafenib, Melanoma, Safety, Japanese patients

## Abstract

**Background:**

Post-approval research or monitoring is important to determine real-world safety of new products; however, evidence is scant for vemurafenib in Japanese patients. In Japan, a unique system is officially obligated to investigate post-approval safety. Here we report the first adverse drug reaction (ADR) data from vemurafenib-treated Japanese patients with metastatic melanoma. Data were collected in an early post-marketing phase vigilance (EPPV) study.

**Methods:**

ADRs were events for which a causal relationship with vemurafenib could not be ruled out or was unknown. ADR data were collected for patients treated with vemurafenib (960 mg bid) between 26 February and 25 August 2015.

**Results:**

Among 95 patients, 46 patients had 118 ADRs (24 serious ADRs in 13 patients). The most common serious ADRs were hypersensitivity (*n* = 1; 3 events), arthralgia (*n* = 2; 2 events), pyrexia (*n* = 2; 2 events) and drug eruption (*n* = 2; 2 events). Seven patients had serious skin disorders or hypersensitivity, six of whom had prior anti-programmed cell death-1 (PD-1) antibodies 5–35 days before starting vemurafenib. ADR reports of serious skin disorders appeared to be collected more rapidly than previously reported. Cutaneous squamous cell carcinoma developed in only one patient.

**Conclusions:**

EPPV in Japanese vemurafenib-treated patients identified no new safety signals. The most serious skin and hypersensitivity ADRs occurred in patients with prior anti-PD-1 exposure. Cutaneous squamous cell carcinoma appeared to be rare in Japanese patients. Further research is needed to clarify whether prior treatment with anti-PD-1 agents or racial differences affect the characteristic profile of cutaneous ADRs in Japanese patients.

**Electronic supplementary material:**

The online version of this article (doi:10.1007/s12094-017-1706-2) contains supplementary material, which is available to authorized users.

## Introduction

Although melanoma is less common in Japan than in other developed countries, such as the US and Australia, the incidence of this condition has been increasing in recent years [1–3]. An estimated 1371 new cases were diagnosed in 2012 [4]; approximately 26–40% of these patients have the *BRAF* ^V600^ gene mutation [5, 6].

Before 2011, treatment options for patients with metastatic melanoma were limited and outcomes were poor [7]. In 2011, the US Food and Drug Administration approved the BRAF kinase inhibitor vemurafenib for the treatment of patients with metastatic melanoma harbouring the *BRAF* ^V600^ mutation [8]. This approval was based on the results of the phase III BRIM3 study in patients with previously untreated *BRAF* ^V600E^ mutation-positive metastatic or unresectable melanoma [9], which demonstrated significantly improved progression-free survival and overall survival in patients treated with vemurafenib compared with dacarbazine, the previous standard of care. Subsequently, other targeted agents have been approved for the treatment of patients with metastatic melanoma, including the MEK inhibitors, cobimetinib [10] and trametinib [11], the anti-cytotoxic T-lymphocyte antigen 4 (CTLA-4) inhibitor ipilimumab [12], and the anti-programmed cell death-1 (PD-1) agents nivolumab [13] and pembrolizumab [14].

Vemurafenib was approved for use in Japan in December 2014. The Japanese risk management plan includes an early post-marketing phase vigilance (EPPV) process, which is a unique system of post-marketing surveillance in Japan conducted during the 6 months after launch for most new active ingredients; similarly, approval of vemurafenib was conditional on performance of EPPV. During EPPV, medical representatives regularly visit medical institutions to collect safety information, in particular, to monitor the emergence of adverse drug reactions (ADRs). Here we report the findings from the vemurafenib EPPV study.

## Patients and methods

### Study design

Patients included in this prospective EPPV study were those who took part in the patient registry surveillance for vemurafenib that was conducted in Japan as a part of a post-approval commitment. All patients planning to use vemurafenib during the enrolment period were subject to registration in the study.

The EPPV study was planned to examine occurrences of ADRs, under the conditions of actual use, at medical institutions in Japan over the 6-month period following the launch of vemurafenib. During this period, medical representatives visited the study sites every 2 weeks and reported any ADRs either using an electronic ADR reporting form, by fax or email. The objective of the study was to provide information on the safety profile of vemurafenib and to detect any new safety signals appearing in real-world clinical practice.

The study was performed in accordance with Good Vigilance Practice of the Ministry of Health, Labour and Welfare, Japan. As this was a regulatory mandated study, patients were not required to provide informed consent; patients provided informed consent for treatment as per standard procedure at the individual institutions.

### Patients

EPPV studies are not generally limited by the inclusion and exclusion criteria; however, patients with a baseline QTc ≥ 500 ms were not eligible for inclusion in line with the Japanese prescribing information for vemurafenib.

### Treatment

Patients received vemurafenib at the usual adult dosage (960 mg bid), based on the approved dosage modified as needed in routine treatment as shown in Table 1. Treatment continued as needed as EPPV studies do not have a predefined observational period. Treatment delays, suspensions, or dose modifications were performed as needed. Dose modifications were performed in cases of an adverse reaction or development of QT prolongation as shown in Table 1.

**Table 1:** Dose modification

Grade (NCI-CTCAE)^a^	Recommended dose modification
Grade 1 or Grade 2 (tolerable)	Dose modification unnecessary
Grade 2 (intolerable) or Grade 3	
1st appearance	Interrupt treatment until recovery to Grade 0 to 1 or baseline, then resume dosing at 720 mg bid^b^
2nd appearance	Interrupt treatment until recovery to Grade 0 to 1 or baseline, then resume dosing at 480 mg bid^c^
3rd appearance	Discontinue permanently
Grade 4	
1st appearance	Discontinue permanently or, if it is preferable to continue treatment for a patient, interrupt treatment until recovery to Grade 0 to 1 or baseline, then resume dosing at 480 mg bid^c^
2nd appearance	Discontinue permanently
Prolongation of QT interval	
QTc >500 ms with >60 ms change from pre-treatment values	Discontinue permanently
QTc >500 ms with ≤60 ms change from pre-treatment values	
1st appearance	Interrupt treatment until recovery to QTc ≤500 ms, then resume treatment at 720 mg bid^b^
2nd appearance	Interrupt treatment until recovery to QTc ≤500 ms, then resume treatment at 480 mg bid^c^
3rd appearance	Discontinue permanently

^a^Graded by National Cancer Institute Common Terminology Criteria for Adverse Events (NCI-CTCAE) v4.0

^b^The dose should be 480 mg if the dose was reduced to 720 mg before treatment interruption

^c^Treatment should be discontinued permanently if the dose was reduced to 480 mg before treatment interruption

### Assessments

Tests performed before treatment included *BRAF* testing of tumour samples (cobas 4800 *BRAF*V600 Mutation Test, Roche Molecular Systems, Branchburg, NJ, USA), dermatological evaluation, head and neck examination, chest computed tomography, electrocardiogram, electrolyte measurement, liver enzyme levels and eye examination. During treatment, patients underwent the same tests routinely with the exception of *BRAF* testing, which was only performed before starting administration of vemurafenib.

### Adverse drug reactions

ADR data were collected as spontaneous reports during the 6-month EPPV period. Clinically significant ADRs were carefully monitored. ADRs were defined as events for which a causal relationship with vemurafenib could not be ruled out or was unknown. A serious ADR was defined as any event that resulted in death, was life-threatening, required inpatient hospitalisation or prolongation of existing hospitalisation for treatment, was equivalent in seriousness to any of the aforementioned events, resulted in persistent or significant disability/incapacity, or resulted in a congenital anomaly/birth defect. In the event of a serious ADR, more detailed information was collected on the patient using a case report form.

Labelled and unlabelled ADRs were collected. Labelled ADRs are those that might be expected based on the prescribing information. Unlabelled ADRs are those for which the nature or severity are not consistent with information in the relevant source document(s), such as the drug’s prescribing information. Until source documents are amended, expedited reporting is required for additional occurrences of the reaction.

The risk management plan for vemurafenib investigated nine important identified risks: cutaneous squamous cell carcinoma (cuSCC), other secondary malignancies, liver injury, photosensitivity, corrected QT interval (QTc) prolongation, skin disorder (e.g. Stevens–Johnson syndrome, toxic epidermal necrolysis), hypersensitivity, eye disorders and potentiation of radiation injury. Four important potential risks were also investigated: progression of *RAS* mutant malignancy, bone marrow depression, facial paralysis and gastrointestinal polyps. These were selected based on the results of clinical studies performed in Japan and elsewhere and events detected in post-marketing experience in countries outside of Japan where vemurafenib was approved.

## Results

### Patient characteristics

According to the patient registry surveillance, 95 patients were treated with vemurafenib in Japan during the EPPV period (26 February–25 August 2015). Of these, 52 patients were male and 43 patients were female; patient mean age was 58.9 years. Detailed information was only collected for patients who developed serious ADRs (*n* = 13; Table 2).

**Table 2:** Demographics and baseline characteristics of patients who developed serious adverse drug reactions (*n* = 13)

Characteristic	Value
Mean age, years (range)	57.5 (24–85)
Male, *n* (%)	9 (69)
ECOG performance status, *n* (%)	
0	7 (54)
1	6 (46)
2	0
Metastatic melanoma stage, *n* (%)	
IIIc	1 (8)
IV	1 (8)
IV (M1b)	2 (15)
IV (M1c)	9 (69)
Treatment line, *n* (%)	
First	1 (8)
Second	3 (23)
Third	6 (46)
Fourth	2 (15)
Unknown	1 (8)
Prior therapy, *n* (%)	
Interferon beta	1 (8)
Nivolumab	9 (69)
Dabrafenib	1 (8)
Pembrolizumab	1 (8)
Unknown	1 (8)
None	1 (8)

At baseline, one patient had QT prolongation with a QTc value <500 ms. Five patients had electrolyte abnormalities that were adjusted before the start of the study and the patients were considered eligible.

### Safety

The total number of ADRs collected during the EPPV period was 118 events in 46 patients. These are summarised in Table 3 and detailed in Supplementary Table 1. The most common ADRs were arthralgia, rash and pyrexia.

**Table 3:** Adverse drug reactions occurring in >1 patient in the Japanese early post-marketing phase vigilance period

Event (*n*)	Serious	Non-serious	All
All events	24	89	113
Arthralgia	2	11	13
Rash	1	7	8
Pyrexia	2	6	8
Myalgia	1	6	7
Drug eruption	2	4	6
Photosensitivity reaction		5	5
Skin disorder	1	3	4
Hypersensitivity	3		3
Alopecia		3	3
Erythema multiforme	1	2	3
PPE		3	3
Decreased appetite		2	2
Bundle branch block right		2	2
Diarrhoea		2	2
Hepatic function abnormal		2	2
Acne		2	2
Erythema nodosum		2	2
Hyperkeratosis		2	2
Malaise		2	2
QT prolonged		2	2
Neutrophil count decreased	1	1	2
Platelet count decreased		2	2

*PPE* palmar–plantar erythrodysesthesia syndrome

A total of 24 serious ADRs were reported in 13 patients. The most common were hypersensitivity (three events in one patient), and drug eruption, pyrexia and arthralgia (two events in two patients for each). Seven serious skin disorders including hypersensitivity reactions were reported, six of which were seen in the patients who had previously been treated with nivolumab or pembrolizumab within the 2 months before starting vemurafenib. Facial paralysis developed in one patient who had also been previously treated with nivolumab. These are summarised in Table 4. One patient with a history of nivolumab treatment experienced three hypersensitivity episodes after administration and re-administration of vemurafenib. The first episode occurred with myalgia, arthralgia, fatigue, and a fever of up to 38 °C and erythemas of various sizes across the patient’s entire body 9 days after starting vemurafenib. A second onset of hypersensitivity developed 2 h after re-administration of vemurafenib, the symptoms of which included hyperemia, periocular skin disorder and mucosal symptoms, which spread systemically, with pyrexia of 39 °C. A third episode of hypersensitivity developed with rash (rose–pink erythema) on the patient’s anterior chest 12 days after re-administration of a reduced dose of vemurafenib. Three episodes of hypersensitivities recurred during prednisolone taper and vemurafenib was discontinued.

**Table 4:** Serious skin disorders and hypersensitivity reactions occurring in patients in the vemurafenib early post-marketing phase vigilance study

Case no.	Events	Prior therapy	Outcome	Re-administration of vemurafenib
1	Skin disorder	Pembrolizumab until Day −28	Recovered	Yes
2	Hypersensitivity^a^	Nivolumab until Day −5	Improved	Yes
3	Rash	Interferon beta	Recovered	Yes
4	Drug eruption	Nivolumab until Day −21	Improved	Yes
5	Stevens–Johnson syndrome	Nivolumab until Day −21	Improved	No
6	Erythema multiforme	Nivolumab until Day −35	Recovered	Yes
7	Drug eruption	Nivolumab until Day −35	Recovered	No

^a^Previously reported by Imafuku et al. [15]. This patient experienced three hypersensitivity episodes

Important identified risks and important potential risks are shown in Table 5. cuSCC, which is one of the most characteristic adverse events of BRAF inhibition, was observed in only one patent in the present study. All events of QTc prolongation that developed in this study were non-serious. One patient had a serious liver disorder (Grade 3). Vemurafenib was subsequently restarted at half the initial dose (480 mg bid) in this patient after improvement of the adverse event. However, vemurafenib was withdrawn for exacerbation of liver toxicity, which resolved 14 days after discontinuation of vemurafenib. One patient who developed a serious unlabelled ADR—hyperkalemia—had a sudden increase in potassium level after vemurafenib administration that was controlled with a potassium chelating agent. This event was followed by tumour regression.

**Table 5:** Important identified risks

Adverse drug reaction	Serious events		All events	
	No. of patients	No. of events	No. of patients	No. of events^a^
Important identified risks				
cuSCC	1	1	1	1
Other second malignancy	0	0	0	0
Liver injury	1	1	5	5
Photosensitivity	0	0	5	5
QTc prolongation	0	0	2	2
Skin disorder	5	5	17	17
Hypersensitivity	2	4	10	12
Eye disorders	0	0	2	2
Important potential risks				
Progression of *RAS* mutant malignancy	0	0	0	0
Facial paralysis	1	1	1	1
Myelosuppression	1	3	3	5
Gastrointestinal polyps	0	0	0	0

*cuSCC* cutaneous squamous cell carcinoma

^a^Serious and non-serious events

## Discussion

Since the approval of vemurafenib for the treatment of patients with metastatic melanoma in 2011, a large body of evidence has been accumulated demonstrating the tolerability of vemurafenib. However, the data are limited to the clinical trial setting and to Caucasian patients. Little evidence exists in real-world use, although post-approval research or monitoring is important in determining the real-world safety of new products. Moreover, evidence of the real-world safety of vemurafenib is scant in Japanese patients. In Japan, there is a unique system (EPPV studies) that is officially obligated to investigate post-approval safety. After the approval of vemurafenib in Japan in 2015, the present EPPV study was performed in line with Japanese regulatory requirements. The aims of this study were to clarify the real-world safety of vemurafenib in Japanese patients and to assess whether racial differences exist between Caucasian and Japanese patients. This EPPV study identified no new safety signals for vemurafenib compared with previous reports from large-scale clinical studies [9, 16], although some differences in cutaneous ADRs were observed in our Japanese patients.

The most common ADRs observed in Japanese patients in the EPPV study were arthralgia and rash. Serious ADRs included hypersensitivity, arthralgia, pyrexia and drug eruption. These were similar to previous reports [9, 16]. However, cuSCC was seen in only one patient and keratoacanthoma was not observed in our study. The previous safety study reported that cuSCC, keratoacanthoma and Bowen’s disease developed in 14% of patients [16]. Because the number of the examined cases is limited in the present study, further examination is required to clarify whether difference in skin type influences the occurrence of vemurafenib-related skin tumours. It is also possible that duration of treatment may be a risk factor in the development of drug-related skin tumours; further investigation of this is also warranted.

Skin reactions were common in patients in this study and included rash, drug eruption, photosensitivity and erythema multiforme in line with previously reported clinical trials [9, 16].

Six of seven patients in the EPPV study who developed serious skin disorders had previously been treated with nivolumab or another PD-1 antibody before receiving vemurafenib. Nivolumab was approved before vemurafenib in Japan, resulting in many patients using nivolumab first, before starting vemurafenib after its approval in February 2015. This is not a comparative study, but these results may suggest that previous treatment with anti-PD-1 antibodies could affect the seriousness of skin disorders if vemurafenib is used after anti-PD-1 agents. Further research is needed to clarify whether prior treatment with anti-PD-1 agents induces more severe skin disorders including hypersensitivity ADRs. The results of the EPPV study may help assess the safety characteristics of vemurafenib when used after anti-PD-1 antibodies.

Other important serious AEs in our study included facial paralysis in one patient. Facial paralysis associated with vemurafenib has been previously reported [17–20]. The patient who developed facial paralysis in our study had previous treatment history of nivolumab and concurrently developed serious muscular weakness, drug eruption (causality of vemurafenib was denied) and non-serious photosensitivity reaction. Because the number of such cases is limited in the present study, it is unclear whether these events were common in Japanese patients.

Some limitations of the present study should be considered. Detailed information was only collected for patients in whom serious ADRs developed. In addition, the observation period was short, which may have limited the number of ADRs observed. Further observation over a longer period may be needed to clarify the safety profile of vemurafenib in Japanese patients.

In conclusion, this EPPV study identified no new safety signals for vemurafenib in Japanese patients with metastatic melanoma; however, some differences compared with previous studies were observed, namely a lower incidence of cuSCC and keratoacanthoma, and higher incidence of serious skin disorders. The most serious skin and hypersensitivity ADRs occurred in patients with prior anti-PD-1 exposure. Further research is needed to clarify whether prior treatment with anti-PD-1 agents or racial differences affect cutaneous ADRs in Japanese patients.

## Electronic supplementary material

Below is the link to the electronic supplementary material.

Supplementary material 1 (DOCX 15 kb)
