# Supplementary material for: Characteristics of adverse drug reactions in a vemurafenib early post-marketing phase vigilance study in Japan
Source: Clin Transl Oncol. 2017 Jul 3;20(2):169–75. doi: 10.1007/s12094-017-1706-2 (PMC5797186; doi:10.1007/s12094-017-1706-2)
Supplement: Supplementary file 1 — Supplementary material 1 (DOCX 15 kb) [file 12094_2017_1706_MOESM1_ESM.docx]

**Supplementary Table 1.** Adverse drug reactions recorded during the Japanese early post-marketing phase vigilance period

| **Events** | **Serious** | **Non-serious** |
| --- | --- | --- |
| All events | 24 | 89 |
| Arthralgia | 2 | 11 |
| Rash | 1 | 7 |
| Pyrexia | 2 | 6 |
| Myalgia | 1 | 6 |
| Drug eruption | 2 | 4 |
| Photosensitivity reaction |  | 5 |
| Skin disorder | 1 | 3 |
| Hypersensitivity | 3 |  |
| Alopecia |  | 3 |
| Erythema multiforme | 1 | 2 |
| PPE |  | 3 |
| Decreased appetite |  | 2 |
| Bundle branch block right |  | 2 |
| Diarrhoea |  | 2 |
| Hepatic function abnormal |  | 2 |
| Acne |  | 2 |
| Erythema nodosum |  | 2 |
| Hyperkeratosis |  | 2 |
| Malaise |  | 2 |
| QT prolonged |  | 2 |
| Neutrophil count decreased | 1 | 1 |
| Platelet count decreased |  | 2 |
| Conjunctivitis |  | 1 |
| Melanocytic naevus |  |  |
| Neoplasm skin |  |  |
| Skin papilloma |  | 1 |
| cuSCC | 1 |  |
| Dehydration |  | 1 |
| Hyperkalaemia | 1 |  |
| Headache |  | 1 |
| VIIth nerve paralysis | 1 |  |
| Ocular hyperaemia |  | 1 |
| Uveitis |  | 1 |
| Abdominal pain lower |  | 1 |
| Faeces pale |  | 1 |
| Nausea |  | 1 |
| Pancreatitis | 1 |  |
| Liver disorder | 1 |  |
| Erythema |  | 1 |
| Intertrigo |  | 1 |
| Milia |  |  |
| Pruritus |  | 1 |
| Stevens–Johnson syndrome | 1 |  |
| Muscular weakness | 1 |  |
| Musculoskeletal stiffness |  | 1 |
| Renal disorder |  | 1 |
| Renal impairment |  | 1 |
| Acute kidney injury | 1 |  |
| Endometrial atrophy |  |  |
| Blood bilirubin increased |  | 1 |
| Blood creatinine increased |  | 1 |
| Lymphocyte count decreased | 1 |  |
| White blood cell count decreased | 1 |  |
| Blood bilirubin abnormal |  | 1 |

cuSCC, squamous cell carcinoma of the skin; PPE, Palmar-plantar erythrodysaesthesia syndrome.
